# A Retrospective Analysis of Clinico-Demographic and Genetic Characteristics and Treatment Outcomes in Isoniazid Mono-Resistant Tuberculosis Patients: A Single-Center Study

**DOI:** 10.7759/cureus.42166

**Published:** 2023-07-19

**Authors:** Parikh K Hymn, Yamini Gurjar, Nikita M Savani

**Affiliations:** 1 Pulmonary Medicine, Shantabaa Medical College and General Hospital, Amreli, IND; 2 Community Medicine, Shantabaa Medical College, Amreli, IND

**Keywords:** tuberculosis, mono-resistant, genetic mutation characteristics, demographic characteristics, clinical characteristics, successful treatment outcome

## Abstract

Introduction: Treatment failure and relapse rates are more likely to occur when there is isoniazid (INH) resistance. So, we can no longer ignore the problem of isoniazid mono-resistance. It is pertinent to control the spread of primary INH resistance and prevent secondary resistance.

Aim: This study aims to evaluate subjects’ clinical, demographic, and genetic characteristics and explore their treatment outcomes.

Methods: All data of isoniazid mono-resistant tuberculosis (TB) patients, which were maintained in the electronic database of mandatory notifications (NIKSHAY Portal) between 2017 and 2022, were reviewed. A total of 54 patients were included after excluding five patients with ongoing treatment.

Results: Of 54 patients, 41 (75.9%) were cured, which was classified under favorable outcome, and the rest were classified under unfavorable outcome. Phenotypic, high-level mutation (*katG*) was found in 48 (88.9%) patients. Kaplan-Meier curves show that survival probabilities increase in weeks with regular intake of drugs.

Conclusion: Our study found that those with younger ages and males were more affected. We found favorable outcomes in the majority of patients.

## Introduction

Recent global data showed that about a half million new cases of rifampicin-resistant tuberculosis (RR-TB) were documented in 2019 with 78% of them having confirmed multidrug-resistant TB (MDR-TB) [1]. According to guidelines for programmatic management of drug-resistant TB (DR-TB) in India, in 2021, there were 124,000 MDR/RR-TB cases in India (9.1/lakh population) [1]. In presumptive DR-TB patients, rifampicin resistance detection is prioritized. Without knowing the isoniazid (INH) status, rifampicin-sensitive patients may be wrongly classified as drug-sensitive TB. Treating such erroneously classified drug-sensitive TB may lead to poor TB outcomes and the emergence of drug-resistant TB [2]. Isoniazid is bactericidal and an important first-line anti-TB drug. Isoniazid mono-resistance, as well as in combination with other drug resistance, is a common type of resistance globally [3].

A national anti-TB drug resistance survey from India showed that isoniazid resistance is 11.06% in new TB cases (confidence interval (CI): 9.97%-12.22%) and 25.09% in previously treated TB cases (CI: 23.1%-27.11%) [4]. Isoniazid mono-resistance has been linked to death in TB meningitis patients as isoniazid is the only bactericidal agent that can cross the blood-brain barrier [5]. In their systematic review and meta-analysis, Menzies et al. [6] showed that initial INH resistance increases the incidence rates of treatment failure and relapse. It is pertinent to control the spread of primary INH resistance and prevent secondary resistance [7].

These data suggest that we can no longer ignore the problem of isoniazid mono-resistance. Hence, studying the isoniazid mono-resistant TB population in the context of diagnosis, clinical, demographic, and microbiological characteristics, and treatment outcomes is much needed. Therefore, with this aim, we evaluated the clinical, demographic, and genetic characteristics of subjects from the Amreli district of Saurashtra region and explored their treatment outcomes.

## Materials and methods

Study design and subject

The Amreli district of Gujarat, India, has 11 TB units (TUs), 59 peripheral health institutions (PHIs), and one district TB center (DTC). DTC is the nodal center for tuberculosis. This retrospective cross-sectional study was conducted among all isoniazid mono-resistance TB patients registered by DTC, Amreli.

According to the National Tuberculosis Elimination Programme (NTEP) guideline, all TB cases should be recorded and registered. Sputum samples should be submitted for acid-fast smear and mycobacterial culture. First-line line probe assay (LPA) was done in all sputum-positive samples. There were 59 isoniazid mono-resistant TB patients registered in the electronic database of mandatory notifications (NIKSHAY Portal) between 2017 and 2022, and all these 59 patients were reviewed for study enrollment.

Exclusion criteria

Those patients who were transferred out (moved to the other treatment unit) within two months of anti-TB treatment and patients with all other types of drug resistance, patient-initiated treatment for MDR-TB, and second-line drugs due to adverse reactions to first-line drugs were reviewed for exclusion, but no such patient was found. However, of 59 patients, five were still on ongoing treatment at the time of analysis, and their outcome was pending, so they were excluded. Thus, a total of 54 subjects were included in the study.

Data tool and procedure

Data was collected from reviewing the medical records and NIKSHAY Portal with the help of DTC, Amreli. We ascertained the demographic, epidemiological, and clinical characteristics of all study subjects using a structured questionnaire. Data obtained associated with possible predicting factors such as age, gender, type of mutation through LPA (*katG*/*inhA*), weight band (25-45 kg/46-70 kg), previous history of anti-tubercular treatment (ATT), history of smoking, HIV status, presence of diabetes mellitus (DM), the occurrence of adverse drug reactions (ADRs) during treatment, and the duration of intensive phase (IP) as determined by sputum conversion (3/4/5/6 months) were retrieved. These variables were examined in terms of the outcome of the treatment. Some of the data that was not in medical records were gathered from patients/patients’ family members through telephone interviews.

Definitions

A TB patient whose biological sample exclusively exhibits resistance to one first-line anti-TB drug is considered to have mono-resistance TB [1]. A TB patient whose biological specimen is resistant to isoniazid and whose susceptibility to rifampicin has been confirmed is considered to have isoniazid resistance TB [1]. Those with TB whose biological specimen is resistant to isoniazid and rifampicin with or without resistance to other first-line drugs are considered to have multidrug resistance TB (MDR-TB) [1]. Treatment failed is defined as a patient whose treatment regimen needs to be terminated or permanently changed to a new regimen option or treatment strategy [1]. Cured patients are those with pulmonary TB with bacteriologically confirmed TB at the beginning of treatment who completed treatment as recommended by the national policy with evidence of bacteriological response and no evidence of treatment failed [1]. Treatment completed is defined as a patient who completed treatment as recommended by the national policy whose outcome does not meet the definition of cure or treatment failed [1]. Died refers to a patient who died before starting or during the course of treatment [1]. Lost to follow-up (LTFU) refers to patients who did not start treatment or whose treatment was interrupted for two consecutive months or more. It may result in unfavorable outcomes such as death, a regimen changed to MDR, or permanent morbidity [1].

Diabetes mellitus is defined as fasting plasma glucose (FPG) ≥ 126 mg/dL or glycated hemoglobin ≥ 6.5%. In previously treated patients, past history of ATT was defined as per Technical Operational Guidelines (TOG) [1].

Line probe assays (LPA) use polymerase chain reaction (PCR) and reverse hybridization methods for the detection of mutation isoniazid associated with drug resistance. First-line LPA detects mutations in the *rpoB* gene for R resistance and in the *katG* gene and *inhA* promoter region for H (and ethionamide (Eto)) resistance. Resistance to a high level is inferred as *katG* and a low level is inferred as *inhA* [1].

Sputum (culture/smear) conversion means that a patient had positive sputum for culture/smear before being treated for TB disease, but after starting treatment, it became negative. Successful sputum conversion is usually defined as at least two consecutive negative cultures/smears [1].

Course of treatment and outcome

Patients were given treatment as per TOG. The total duration of treatment ranged from nine to 12 months. The intensive phase (IP) was for three months and extendable up to a maximum of six months as per requirements. Treatment outcomes were defined as per TOG guidelines and categorized into cured completed treatment, died, and lost to follow-up (LTFU). Both cured patients and those with completed treatments were defined as treatment success, whereas failure, death during treatment, and LTFU were defined as unfavorable outcomes. Relapse within two years of finishing treatment was evaluated by a national database of registered users [8].

Statistical analysis

The collected data were entered into Microsoft Office 2021 (Microsoft Corp., Redmond, WA, USA) and analyzed using the statistical software Jamovi version 2.3.26 [9]. Patient characteristics were analyzed descriptively using frequencies and percentages. Kaplan-Meier survival analyses for disease-free survival were constructed, and subgroups (sputum conversion and drug adherence) were compared using the log-rank test. In the log-rank test, P < 0.05 was considered statistically significant.

Ethical consideration

This study was approved by the institutional ethics committee of Shantabaa Medical College, Amreli, with reference number SMC/IEC/45/05/23.

## Results

**Figure 1:**
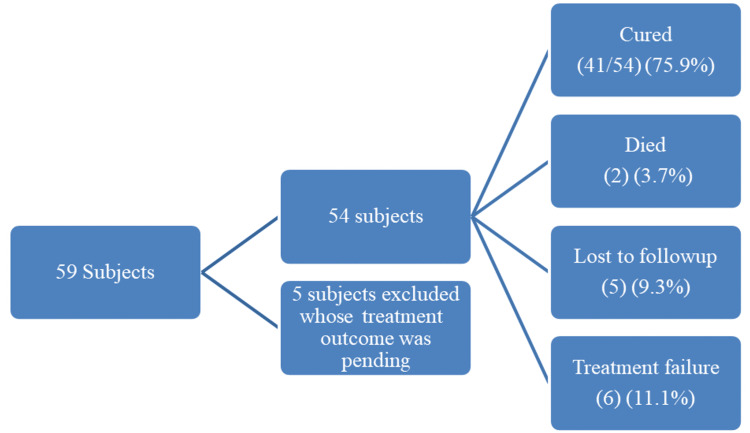
Treatment outcomes of isoniazid mono-resistant patients

A total of 59 subjects were screened, of which 54 were part of the study. Five subjects with ongoing treatment and outcomes that are yet to come were excluded from the study. Of the 54 subjects, 41 (75.9%) were cured, which were classified under favorable outcomes, and the rest were classified under unfavorable outcomes (Figure 1). 

The baseline characteristics of the mono-H-resistant patients are shown in Table 1. The median age was 35.5 (interquartile range (IQR): 22.0-45.7) years, and 37 (68.5%) were male. The majority of them were Hindu (92.6%). More than half of the subjects were laborers (59.3%) and from urban localities (72.2%). The proportion of literate and illiterate was almost similar. Two-thirds of the subjects were married. Of the subjects under study, 19 (35.2%) had an addiction history, of which one (1.9%) had a habit of smoking, 16 (29.6%) had a habit of tobacco smoking, and two had a habit of both tobacco smoking and smoking.

**Table 1:** Baseline characteristics of mono-H-resistant patients (n=54)

Variables		Frequency (number)	Percentage (%)
Age	≤30	24	44.4
	>30	30	55.6
Gender	Male	37	68.5
	Female	17	31.5
Religion	Hindu	50	92.6
	Muslim	4	7.4
Occupation	Businessman	1	1.9
	Farmer	5	9.3
	Housewife	8	14.8
	Job	1	1.9
	Laborer	32	59.3
	Student	4	7.3
	Unemployed	3	5.6
Locality	Rural	15	27.8
	Urban	39	72.2
Education	Illiterate	24	44.4
	Primary	18	33.3
	Secondary	6	11.1
	Higher secondary	6	11.1
Marital status	Married	36	66.7
	Unmarried	18	33.3
Addiction	Yes	19	35.2
	No	35	64.8

A total of 45 (83.3%) patients have sputum conversion. Phenotypic, high-level mutation (*katG*) was found in 48 (88.9%) patients, and low-level mutation (*inhA*) was found in six (11.1%). The number of TB episodes is shown in Table 2, which shows that the first-time occurrence of TB means the first episode and likewise. A family history and past history of TB were found in 31 (57.4%) and 32 (59.3%) patients, respectively. The HIV statuses of all subjects were non-reactive. Regarding intake of drugs, 44 (81.5%) were taking treatment regularly. Diabetes was found in less proportion. Very few subjects have side effects such as joint pain, nausea, and vomiting. The majority of the subjects were given <45 kg weight band of the treatment regimen. Regarding outcome, the majority (41, 75.9%) have a favorable outcome (Table 2).

**Table 2:** Clinical and microbiological profile of mono-H-resistant patients (n=54) TB: tuberculosis

Variables		Frequency (number)	Percentage (%)
Sputum conversion	Yes	45	83.3
	No	9	16.7
Type of mutation	*inhA* (low level)	6	11.1
	*katG* (high level)	48	88.9
Number of TB episodes	>2	9	16.7
	1	22	40.7
	2	23	42.6
Family history of TB	Yes	31	57.4
	No	23	42.6
Past history of TB	Yes	32	59.3
	No	22	40.7
Regular intake of drug	Yes	44	81.5
	No	11	18.5
Diabetes mellitus	Yes	7	13
	No	47	87
Side effects of drug	Yes	4	7.4
	No	50	92.6
Weight band of treatment regimen	16-29 kg	1	1.9
	26-45 kg	9	16.7
	30-45 kg	28	51.9
	46-70 kg	15	27.8
	>70 kg	1	1.9
Treatment outcome	Cured	41	75.9
	Died	2	3.7
	Lost to follow-up	5	9.3
	Treatment failure	6	11.1

**Figure 2:**
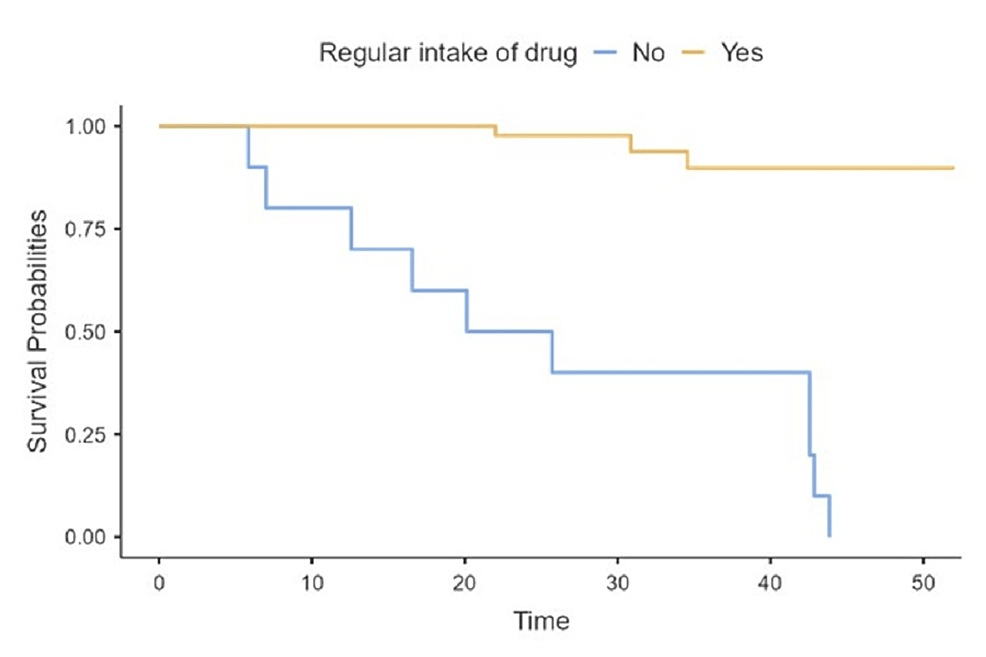
Kaplan-Meier plots and log-rank test for the probability of unfavorable outcomes, according to regular intake of drug

**Figure 3:**
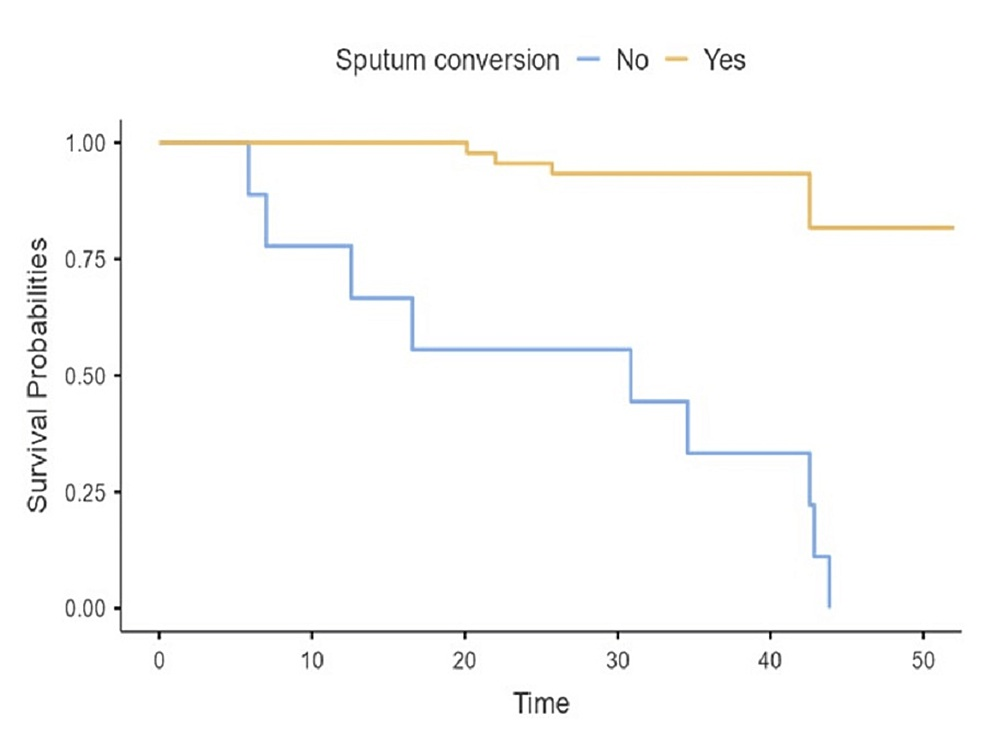
Kaplan-Meier plots and log-rank test for the probability of unfavorable outcomes, according to sputum conversion

Kaplan-Meier analysis and log-rank test revealed that the probability of unfavorable outcomes was significantly different between those with and without sputum conversation (P < 0.001) and between those who take regular drugs and those who do not (P < 0.001) (Figures 2, 3). The Kaplan-Meier curves show that survival probabilities increase in weeks with regular intake of drugs.

## Discussion

Isoniazid is an early bactericidal drug on rapidly proliferating cells. It is a cornerstone drug for an anti-TB regimen. Isoniazid is used in combination with other drugs used to prevent resistance to other anti-TB drugs.

In our study, the median age was 35.5 (interquartile range (IQR): 22.0-45.7) years. Shah et al. [10] also found that the mean age was 28.43 ± 14.32 years in drug-resistant TB. Khan et al. [11] also showed a mean age of 26 years. It has been well-documented that drug resistance to TB occurred in young people. Prior history of TB in our study was seen in 87% (n = 47), which is similar to other studies [12-14].

In the present study, males were more affected (37, 68.5%). In the study of Surkova et al. [15], 70.67% were male and 29.33% were female (P < 0.001), which was comparable to our results. Villegas et al. [16] (2016) also showed similar gender distribution patterns, i.e., 82.1% were male and 17.9% were female.

In the present study, the most common genetic mutation found is *katG *(88.9%). Similarly, Yao et al. (Chongqing, China) [17] observed that of 50 INH mono-resistant patients, 41 (82%) had *katG *mutations and nine (18%) had *inhA *mutations. Huyen et al. (southern Vietnam) [18] concluded that 75.3% had mutations in *katG *and 28.2% had mutations in the *inhA *promoter region. A study by Kigozi et al. (Uganda) [19] observed that 80% had *katG *mutations and 6% had *inhA *mutations. Tavakkoli et al. (Iran) [20] also reported that in 17.24% and 82.76% of the strains, *inhA* genes and *katG* genes, respectively, were responsible for INH resistance. Alagappan et al. [21] in their meta-analysis documented that 1,821 (11.8%) of 15,438 INH-resistant strains had detectable mutations: 71% in *katG* and 29% in the *inhA* promoter region. Mutation in *katG* or *inhA* is clinically relevant to treating physicians [22]. A high degree of resistance to INH is considered when *katG* mutation with or without *inhA* mutation is confirmed. The addition of a high dose of INH will not be effective in the regimen. A mutation limited only to *inhA* is mostly associated with a low degree of INH resistance, and these patients will likely benefit from high doses of INH (10-15 mg/kg/day) [23].

We did not find any statistically significant association (on applying the chi-square test) with smoking status, diabetes, weight band, marital status, adverse drug reaction, and education with isoniazid mono-resistant TB.

Limitation

Our study is retrospective in nature. We have limited subjects registered from one district. So, we were unable to prove statistically the factors responsible for favorable and unfavorable outcomes.

## Conclusions

In our study, we found that *katG* is the most common genetic mutation (88.9%). We also found that those with younger ages and males were more affected. The majority of patients have favorable outcomes. It is important to find isoniazid resistance, and treatment for the prevention of drug resistance to TB should be promptly initiated. Our study had a similar outcome in line with other national and international studies. We reinforce for the detection of isoniazid resistance and genetic analysis for better outcomes and prevention of relapse. This data will be helpful for future planning of TB elimination and control strategies in the nation.
